# Unveiling the Broad Substrate Specificity of Deoxynivalenol Oxidation Enzyme DepA and Its Role in Detoxifying Trichothecene Mycotoxins

**DOI:** 10.3390/toxins16030136

**Published:** 2024-03-05

**Authors:** Yan Zhu, Edicon Tze Shun Chan, Nadine Abraham, Xiu-Zhen Li, Weijun Wang, Lili Mats, Honghui Zhu, Jason Carere, Ting Zhou

**Affiliations:** Guelph Research and Development Centre, Agriculture and Agri-Food Canada, Guelph, ON N1G 5C9, Canadanabrah02@uoguelph.ca (N.A.); lili.mats@agr.gc.ca (L.M.);

**Keywords:** DepA, mycotoxin, 15-acetyl-DON, PQQ-dependent enzyme, substrate specificity, structure–activity relationship, binding mode

## Abstract

DepA, a pyrroloquinoline quinone (PQQ)-dependent enzyme isolated from *Devosia* mutans 17-2-E-8, exhibits versatility in oxidizing deoxynivalenol (DON) and its derivatives. This study explored DepA’s substrate specificity and enzyme kinetics, focusing on DON and 15-acetyl-DON. Besides efficiently oxidizing DON, DepA also transforms 15-acetyl-DON into 15-acetyl-3-keto-DON, as identified via LC-MS/MS and NMR analysis. The kinetic parameters, including the maximum reaction rate, turnover number, and catalytic efficiency, were thoroughly evaluated. DepA-PQQ complex docking was deployed to rationalize the substrate specificity of DepA. This study further delves into the reduced toxicity of the transformation products, as demonstrated via enzyme homology modeling and in silico docking analysis with yeast 80S ribosomes, indicating a potential decrease in toxicity due to lower binding affinity. Utilizing the response surface methodology and central composite rotational design, mathematical models were developed to elucidate the relationship between the enzyme and cofactor concentrations, guiding the future development of detoxification systems for liquid feeds and grain processing. This comprehensive analysis underscores DepA’s potential for use in mycotoxin detoxification, offering insights for future applications.

## 1. Introduction

Trichothecenes are tricyclic sesquiterpenes that encompass more than 200 mycotoxins [1]. Type B trichothecenes, such as nivalenol (NIV), deoxynivalenol (DON) and its acetylated derivatives 3-acetyl-DON and 15-acetyl-DON, present a significant economic impact and health hazard due to their occurrence in the food/feed chains. According to survey data from the Ontario Ministry of Agriculture, Food, and Rural Affairs (OMAFRA) covering the past decade (2013–2022), DON contamination levels in Ontario corn samples surpassed the Canadian regulatory limit of 2 ppm, on average, in 7.9% of cases, with a range from 1% to 18% [2,3]. The severity of contamination varied from year to year, mainly due to the fluctuations in weather conditions in the growing season. Although the presence of trichothecene mycotoxins, including DON, 3-acetyl-DON, 15-acetyl-DON, and NIV, varies based on geographical location, climatic conditions, and agricultural practices, many investigations indicated that these mycotoxins do not occur in isolation but often co-exist, presenting a compounded risk to human and animal health.

**Figure 1:**
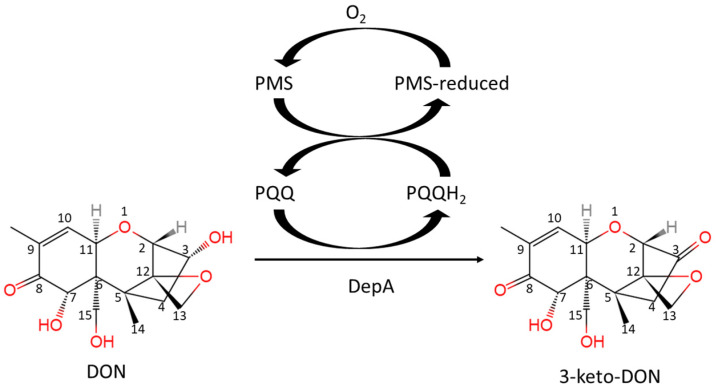
The reaction of DON oxidation catalyzed by DepA with the cofactors of PQQ and PMS.

Current chemical and physical methods are insufficient for reducing trichothecene levels in grains and feed; however, employing microorganisms and their enzymes for biological detoxification offers a more promising strategy [4]. Unearthing and exploiting DON detoxification microorganisms and enzymes continues to be an active area of mycotoxin research. The identification of the DON degrading strain *Devosia mutans* 17-2-E-8 and its associated enzymes DepA and DepB holds promise for future industrial applications. During epimerization, the C3-OH of DON is oxidized by DepA, a Type I PQQ-dependent alcohol dehydrogenase (Figure 1). This produces the less toxic intermediate 3-keto-DON, which is stereo-specifically reduced to 3-*epi*-DON by the second enzyme DepB [5].

15-acetyl-DON is the predominant acetylated DON derivative observed in regions of eastern Canada, like Ontario and Quebec [6]. In terms of its toxicity, studies suggest that 15-acetyl-DON may be more potent than DON, as it impairs intestinal barrier function and reduces proliferation of intestinal epithelial cells [7]. At the molecular level, the general mode of trichothecene toxicity occurs via protein synthesis inhibition [8,9]. The key structural elements of trichothecenes that modulate toxicity include the 12,13-epoxide ring, the double bond between C9 and C10, and the C3-OH group. The biotransformation of the C3-OH group has several implications for the reduced toxicity of DON and 15-acetyl-DON, as well as their biotransformation products 3-keto-DON and 3-*epi*-DON and 3-keto-15-acetyl-DON [10].

Significant studies have been conducted about DON degradation. However, the biotransformation of NIV and the co-occurrent derivatives 3-acetyl-DON and 15-acetyl-DON have been neglected. This gap in knowledge limits our understanding of the full potential of DepA in mycotoxin detoxification. Our study addresses this gap by investigating DepA’s substrate range and the structural basis of its specificity and activity, providing insights into its role in the detoxification process and potential industrial applications.

## 2. Results

### 2.1. DepA Enzyme Activities towards Multiple Mycotoxins

**Figure 2:**
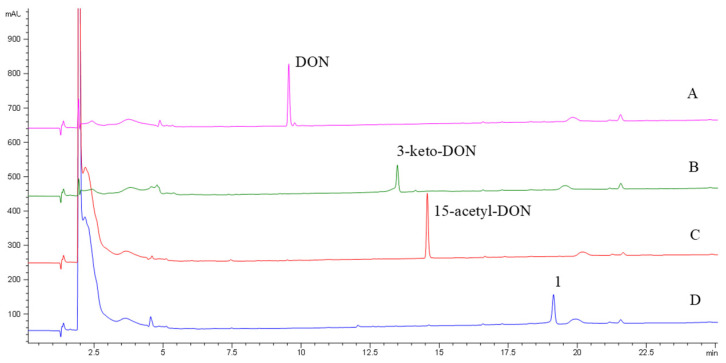
HPLC-DAD chromatograms of DON (**A**), 15-acetyl-DON (**C**) and their transformation products of 3-keto-DON (**B**) and compound **1** (**D**) after treatment via the DepA system.

The DepA enzyme system with cofactors PQQ and phenazine methosulfate (PMS), fully converted from DON and 15-acetyl-DON to 3-keto-DON and an unknown novel product **1,** respectively (Figure 2). The retention times of 3-keto-DON and compound **1** were 12.7 min and 19.1 min, respectively. No enzymatic transformation was observed for NIV and 3-acetyl-DON after 24 h of incubation at room temperature.

### 2.2. Identification of the Biotransformation Product of 15-Acetyl-DON

**Figure 3:**
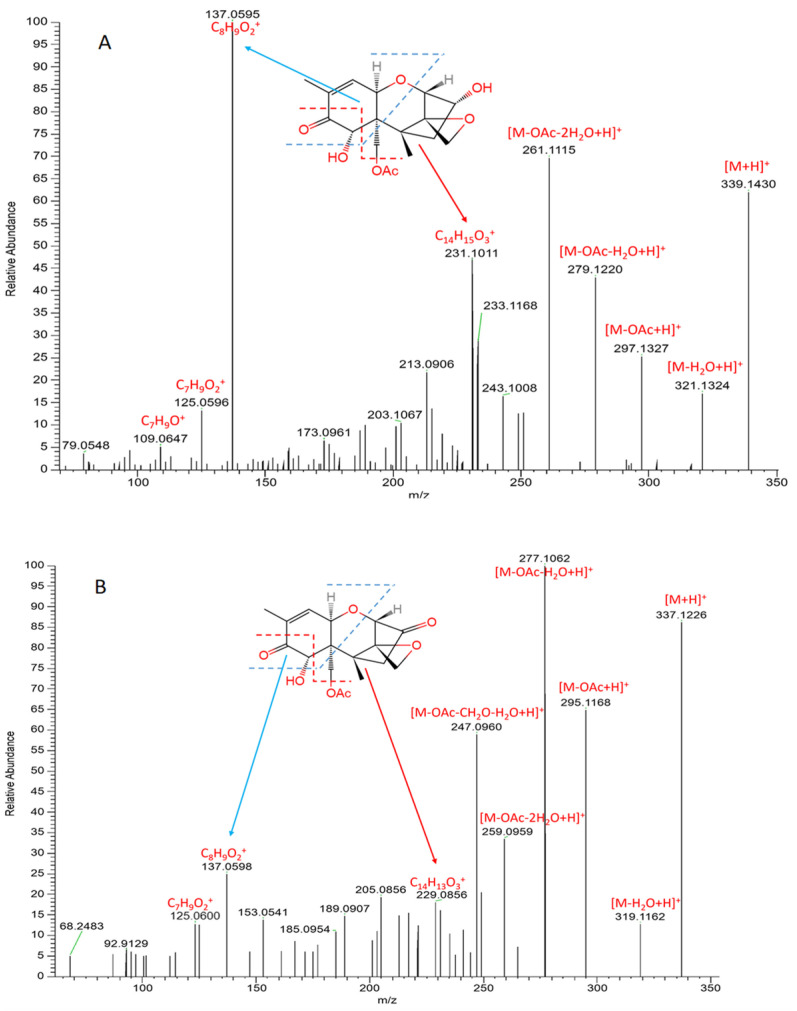
MS/MS spectra of 15-acetyl-DON (**A**) and its biotransformation product (compound **1**) (**B**) after treatment via the DepA system.

The MS/MS analyses of the standard 15-acetyl-DON and its transformation product (compound **1**) are presented in Figure 3. The mass differences of the ions ([M + H]^+^, [M-H_2_O + H]^+^, [M-OAc + H]^+^, [M-OAc-H_2_O + H]^+^, [M-OAc-2H_2_O + H]^+^) between 15-acetyl-DON and compound **1** were 2.0204, 2.0162, 2.0159, 2.0194, and 2.0156, respectively, suggesting a loss of two hydrogen atoms in compound **1** in comparison to 15-acetyl-DON. Furthermore, the same mass decrease was observed in the ion [C_14_H_15_O_3_]^+^ in 15-acetyl-DON (Figure 3), resulting in the ion [C_14_H_13_O_3_]^+^ identified in compound **1**. Proposed cleavages at the bonds of C6-C7, C8-C9, and C15-O indicate that the 2H loss was a result of the oxidation of the hydroxyl group at site C3 rather than C7.

**Figure 4:**
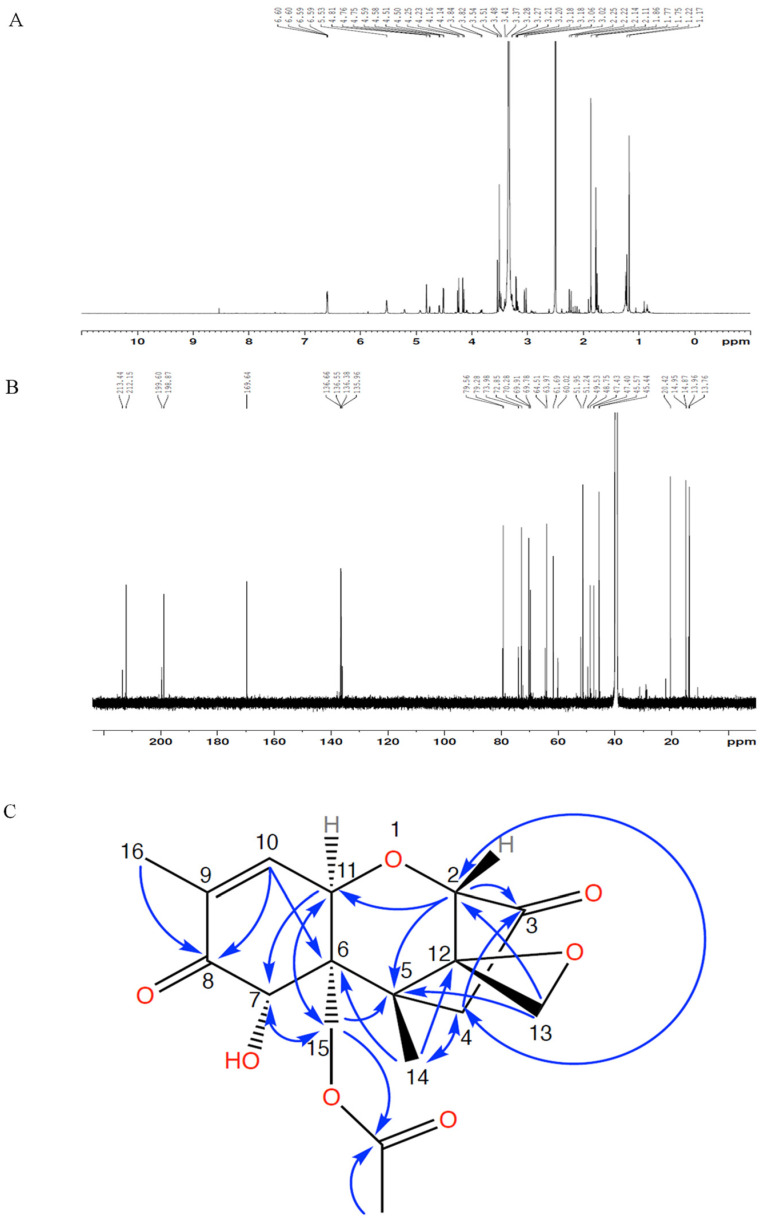
NMR spectra of purified compound **1** ((**A**) ^1^H NMR; (**B**) ^13^C NMR) and HMBC correlations (**C**).

The ^1^H, ^13^C NMR spectra and Heteronuclear Multiple Bond Correlation (HMBC) correlations are presented in Figure 4. The chemical structure of compound **1** was determined via long-range HMBC correlations and comparison of chemical shifts (Table 1) to the 15-acetyl-DON standard. The acetyl group was indicated by ^13^C peaks at 169.6 and 20.4 ppm and a ^1^H peak at 1.86 ppm. The presence of the ketone at position 3 was indicated by a ^13^C peak at 212.3 ppm in compound **1**, which shared a chemical shift of 212.0 ppm in 3-keto-DON similar to that reported by Völkl et al. [11] (Table 1). A minor component (compound **2**) was observed via ^1^H and ^13^C NMR. The compound lacks ^1^H and ^13^C peaks representing the acetyl group but has a ^13^C peak at 213.4 ppm, which matched the reported chemical shifts of 3-keto-DON (Table 1). The presence of 3-keto-DON in the sample is likely due to the degradation of compound **1** in the process of vacuum concentration as the 3-keto-DON was not detected after the DepA treatment.

With the combination of HPLC, LC-MS/MS, and NMR analyses, compound **1** was confidently identified as 3-keto-15-acetyl-DON.

### 2.3. Kinetics of DepA towards DON and 15-Acetyl-DON

The kinetics data of DepA are listed in Table 2. DON displayed significantly greater (*p* < 0.05) V_max_ (14.79 µM·min^−1^) and k_cat_ (1.69 s^−1^) than 15-acetyl-DON (5.99 µM·min^−1^ and 0.69 s^−1^, respectively). Since k_cat_ represents the number of catalytic cycles that each active site undergoes per unit time when the enzyme is saturated with the substrate, the result suggests that DepA catalyzes a faster reaction to oxidize DON than 15-acetyl-DON after binding between the enzyme and the substrates. Conversely, a lower K_m_ toward 15-acetyl-DON was observed, indicating a higher binding affinity relative to DON. In the DepA system, the enzyme showed similar catalytic efficiency for both DON and 15-acetyl-DON (7645 M^−1^s^−1^ and 6389 M^−1^s^−1^, respectively).

### 2.4. Effects of Cofactors on DepA Activity

**Figure 5:**
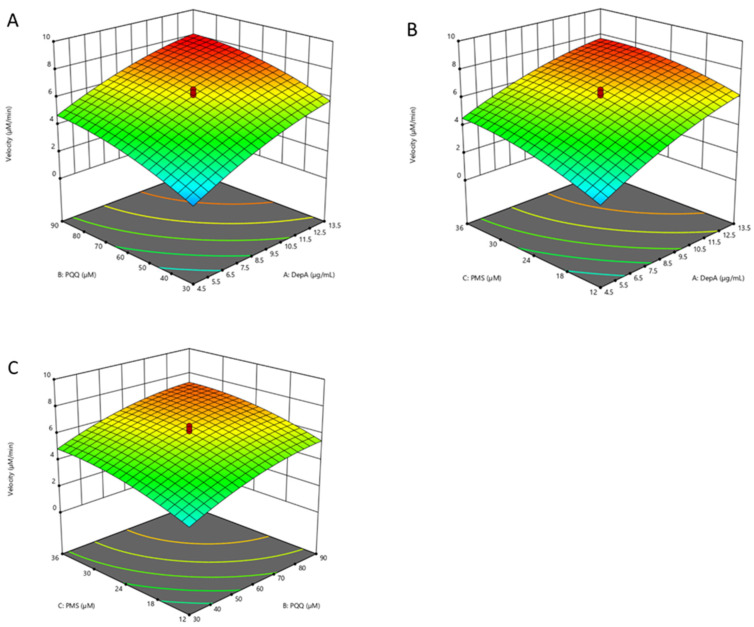
Three-dimensional response surfaces and counter plots illustrating the combined effects of DepA (4.5–13.5 µg·mL^−1^), PQQ (30–90 µM), and PMS (12–36 µM) on the biotransformation rate of DON in the DepA system: (**A**) concentration of DepA and PQQ; (**B**) concentrations of DepA and PMS; (**C**) concentrations of PQQ and PMS.

Using response surface methodology (RSM) combined with central composite rotational design (CCRD), the reaction rates catalyzed by DepA in buffer were determined based on the linear decrease in the substrate (i.e., 50 µg·mL^−1^ of DON) in the first 20 min with 20 different treatment combinations (Figure 5). The reaction rates ranged from 1.051 to 7.904 µM/min under the experimental condition. The data were fitted in a quadratic model (F value = 35.88) that generated a predictive response equation (adeq. precision = 21.80) as Equation (1). X_1_, X_2_, and X_3_ represent the coded values of the concentrations of DepA (µg·mL^−1^), PQQ (µM), and PMS (µM), respectively. The model is significant (*p* < 0.001) according to the ANOVA for the quadratic model.

Reaction rate (µmol·L^−1^·min^−1^) = 6.14 + 1.76X_1_ + 1.25X_2_ + 0.946X_3_ − 0.078X_1_X_2_ − 0.202X_1_X_3_ − 0.52X_2_X_3_ − 0.458X_1_^2^ + 0.523X_2_^2^ − 0.492X_3_^2^ (1)

### 2.5. The Catalytic Mechanism-Assisted Auto-Docking of Different Trichothecenes into the Depa Catalytic Binding Site

**Figure 6:**
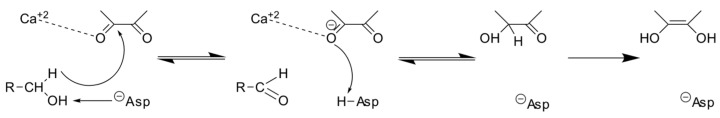
The hydride transfer mechanism of PQQ-dependent dehydrogenases [13]. The hydride-donating carbon atom and PQQ C5 atom are in proximity. Meanwhile, the hydroxyl is in the hydrogen bond range of the catalytic base.

The trichothecenes DON, NIV and 15-acetyl-DON were docked using AutoDock Vina. The DepA–PQQ structure (PDB: 7WMK) was used to create a homology model and its missing loop was modelled using SWISS-MODEL, as described by Yang et al. [12]. Promising orientations were selected based upon highest free binding energy and optimal position for the putative hydride transfer catalytic mechanism (Figure 6). The trichothecenes DON, NIV, and 15-acetyl-DON had the following free binding energies: −7.6 kcal/mol, −7.7 kcal/mol, and −8.1 kcal/mol, respectively. The most optimal configurations among these three trichothecenes all adopt a similar pose in the binding pocket, with the C3-OH within the hydrogen bonding distance to the catalytic aspartic acid.

### 2.6. The Binding Mode of Biotransformed Trichothecenes Provides Insights into Their Reduced Toxicity

**Figure 7:**
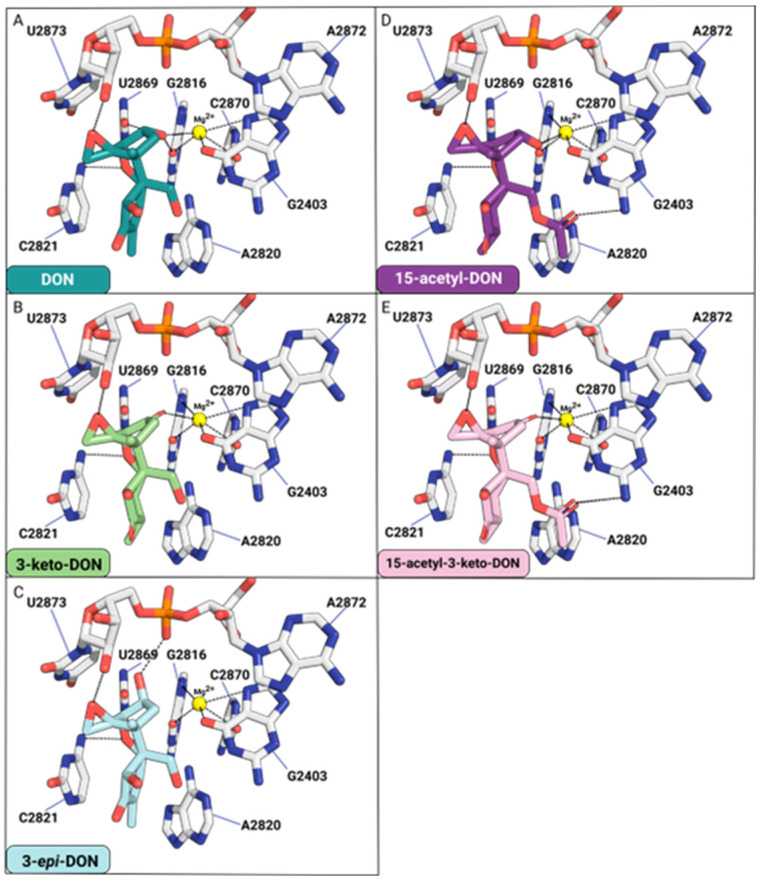
The effects of C3-OH modification for DON (**A**), 3-keto-DON (**B**), 3-*epi*-DON (**C**), 15-acetyl-DON (**D**), and 15-acetyl-3-keto-DON (**E**) binding to the yeast 80S ribosome.

Modifications to the C3-OH group of DON (Figure 7A) and its acetylated derivatives, such as 15-acetyl-DON, can impair binding to the yeast 80S ribosome. The oxidation of the C3-OH group of DON to produce 3-keto-DON obliterates hydrogen bond contacts, specifically with the O2 of uracil U2869 and O6 of guanine G2816 relative to DON (Figure 7B).

For 3-*epi*-DON, the distance between C3-O and Mg^2+^ will increase from 2.7 Å (DON) to 4.5 Å, impairing the interaction required for metal ion coordination. Similarly, losses of two hydrogen bonds between C3-O and O2-G2816 and C3-O and O2-U2869 are observed. The distance between C3-O and O2-G2816 will increase from 3.2 Å (DON) to 5.1 Å (3-*epi*-DON). In contrast, the distance between C3-O and O2-U2869 will increase from 3.2 Å (DON) to 3.4 Å (3-*epi*-DON). Additionally, one hydrogen bond is found between the C3-O (3-*epi*-DON) and the P=O of the phosphate group of U2873 (Figure 7C). Overall, the binding contacts donated by the C3-OH of 3-*epi*-DON will be compromised relative to DON.

15-acetyl-DON differs from DON based on a single acetyl group affixed at the C15 position. Much like DON, the C3-O is involved in Mg^2+^ coordination and hydrogen bonding with the O6 of G2816 (Figure 7D). The acetyl affixed at the C15-OH forms a hydrogen bond contact with the N2 of guanine G2403. This interaction differs from that of DON, which forms hydrogen bond contacts between the C15-OH and ordered water molecules in this pocket. With 3-keto 15-acetyl-DON, the hydrogen bond contact with the O6 of G2816 is eliminated (Figure 7E).

## 3. Discussion

In the field of biological solutions used to detoxify DON, enzymatic detoxification continues to be a growing area of interest. DepA was classified as a quinoprotein (PFAM designation PF13360) and shares a 90.93% sequence identity with the quinoprotein alcohol dehydrogenase (PQQ-ADH) (PDB ID# 4CVB) from *Pseudogluconobacter saccharoketogenes*. In *D. mutans* 17-2-E-8, DepA oxidizes the C3-OH of DON to produce 3-keto-DON using the cofactor PQQ [5,14]. This cofactor is central to the catalytic mechanism shared by other PQQ-dependent enzymes such as galactose oxidase, glucose dehydrogenase, sorbose dehydrogenase, pyranose dehydrogenase, methanol dehydrogenase, and alcohol dehydrogenase [15,16,17,18,19,20].

It is generally accepted that catalysis in most PQQ-dependent dehydrogenases functions via the hydride transfer mechanism (Figure 6) [13]. PQQ is subsequently reduced to pyrroloquinoline quinol (PQQH_2_), which is re-oxidized through electron transfer to physiological electron acceptors in vivo, such as cytochrome cL or cytochrome cGJ in the case of methanol dehydrogenases [21]. In vitro, PMS can be applied as an artificial electron acceptor to regenerate PQQ and facilitate the reaction catalyzed by the PQQ-dependent enzymes. RSM analysis indicates the importance of optimizing the three parameters in the DepA system (i.e., DepA, PQQ, and PMS) to maximize enzyme reaction rates. The methodology could be further applied to the more complex matrix such as liquid animal feed with the benefit of reducing the material cost by optimizing the combination of the enzyme and cofactors.

DepA was determined to possess activity toward 15-acetyl-DON but not 3-acetyl-DON nor NIV. The enzyme possesses a 1.2-fold higher catalytic efficiency towards DON relative to 15-acetyl-DON. To rationalize the observed differences in DepA activity toward these trichothecene substrates, docking analysis was performed using Autodock Vina.

**Figure 8:**
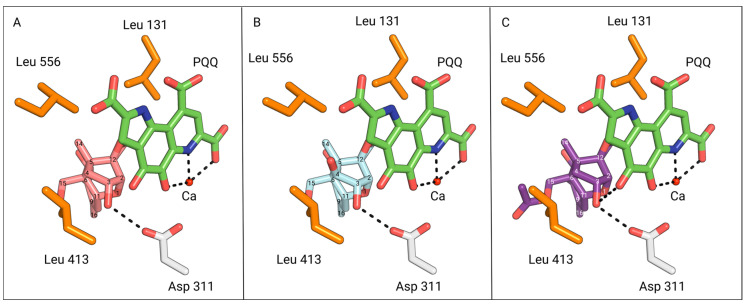
View of DON (pink), NIV (cyan), and 15-acetyl-DON (purple) from the binding pocket outward, oriented in the most optimal catalytic configuration generated via AutoDock Vina. The PQQ cofactor is coloured in green. (**A**) The most optimal configuration for DON based on PQQ catalysis. The C3 hydroxyl is 3.0 Å away from the catalytic Asp-311 residue (white) of DepA. (**B**) The most optimal configuration for NIV based on PQQ catalysis. The C3 hydroxyl is 3.0 Å away from the catalytic Asp-311 residue of DepA. The C3 atom is 3.7 Å away from the PQQ C5 atoms. The C4 hydroxyl of NIV is 3.4 Å and 3.8 Å away from the hydrophobic residues Leu-556 and Leu-413, respectively. (**C**) The most optimal configuration for 15-ADON based on PQQ catalysis. The C3 hydroxyl is 3.0 Å away from the catalytic D311 residue of DepA.

The most optimal docking configuration for DON has a free binding energy of −7.6 kcal/mol, where the proximity is close enough to facilitate hydrogen bond formation between the C3 hydroxyl and catalytic aspartate residue (Figure 8A). The C3 atom is axially positioned within a distance of 3.8 Å to the PQQ C5 atom, allowing for efficient hydride transfer.

Experimental evidence from our study substantiates the activity of 15-acetyl-DON with DepA. This finding is further supported by the optimal AutoDock Vina configuration for 15-acetyl-DON, with a binding energy of −8.1 kcal/mol, and in alignment with the optimal DON configuration (Figure 8C). In both the DON and 15-acetyl-DON optimal configurations, it is noteworthy that the C15 functional group is oriented away from the binding pocket. Based on this orientation, it is anticipated that DON C15 analogs other than 15-acetyl-DON would also adopt a similar configuration and exhibit activity with DepA.

The optimal NIV configuration also mirrors the orientation of the most favourable DON configuration, with the C3 hydroxyl positioned 3.0 Å from the catalytic aspartate residue, within the hydrogen bonding distance (Figure 8B). In addition, the PQQ C5 atom is in close proximity, being 3.7 Å away from the NIV C3 atom.

However, a critical aspect to consider is the substrate’s potential need for rotational flexibility, especially since the 3.7 Å distance approaches the outer limit range required for hydride transfer to PQQ. This flexibility would aid the alignment of the C3 atom with the PQQ C5 axis, effectively shortening the hydride transfer distance. Although DON and NIV share the same configuration, it may be worthwhile to investigate the impact of restricted movement caused by the pocket size and hydrophobic lip as a potential contributing factor to its inactivity. The presence of the hydrophobic leucine residues would possibly hinder rotational flexibility and constrain the pocket size around the substrate’s C4 functional group (Figure 8B). In conjunction with experimental data indicating DepA’s inactivity against NIV, it is plausible that analogs with a C4 functional group similar to or larger than a hydroxyl are also unlikely to display activity.

It is important to acknowledge that docking analysis may not fully capture the dynamic nature of ligand binding. This limitation is heightened even more by the presence of a dynamic loop in the DepA-PQQ complex structure (PDB: 7WMK), which was replaced with a static loop for docking analysis. The dynamic nature of DepA, predominantly composed of fluctuations in the mobile loop, introduces more complexity that has the potential to alter free binding energy due to the loop’s proximity to the binding site. As a result, configurations proposed by docking software programs may not entirely align with the reality of the ligand–protein interaction. In light of this fact, the integration of experimental evidence and computational docking analysis demonstrated an ability to explore the substrate and structural dynamics of DepA to provide a comprehensive assessment of its enzymatic capabilities.

Various chemotypes of *Fusarium graminearum* have the capacity to produce DON derivatives through deacetylation at either the C-3 or C-15 position of 3,15-diacetyl-DON, forming 15-acetyl-DON or 3-acetyl-DON, respectively [22]. In vitro cytotoxicity assays in many cell models suggested a similar or higher toxicity of 15-acetyl-DON than DON [23,24,25]. Moreover, the co-occurrence of DON and its derivatives may act synergistically to cause more harm to human health, although the incidence of 15-acetyl-DON in DON-contaminated cereals has been shown to be low [26]. In silico docking analysis of the 15-acetyl-DON biotransformation product 15-acetyl-3-keto-DON into the 80S ribosome suggests that the oxidation of the C3-OH destabilizes binding due to the loss of two hydrogen bond contacts and impaired metal ion coordination to Mg^2+^. Consequently, the results suggest that 15-acetyl-3-keto-DON will possess reduced toxicity.

## 4. Conclusions

The investigation of the substrate specificity of DON oxidation enzyme DepA reveals multiple mycotoxin detoxification activities and potentially wide applications. The 15-acetyl-3-keto-DON, a biotransformation product of 15-acetyl-DON, was first identified via LC-MS/MS and NMR analysis. The structure–activity relationship analysis based on the binding mode between the enzymatic transformation products and the 25S rRNA in *Saccharomyces cerevisiae* ribosome predicts the decreased toxicity of 15-acetyl-3-keto-DON. Moreover, the mathematics model established based on the correlation between the enzymatic reaction rate and the enzyme and its cofactors describes the efficiency of the DepA enzyme system, which will guide further application studies, including those in the liquid feed or grain-processing matrixes.

For future research, validating the reduced toxicity of the transformation product through in vitro cell models or in vivo animal trials and exploring DepA’s practical applications in real-world agricultural settings will be critical steps forward. These efforts could pave the way for more effective mycotoxin detoxification strategies in food safety and agriculture.

## 5. Materials and Methods

### 5.1. Enzymes and Chemicals

The enzyme catalyzing DON oxidation (i.e., DepA) was identified in *Devosia mutants* 17-2-E-8 and transferred into *E. coli* BL21 via vector pET28a. The purification of DepA was carried out according to the reported protocol [5]. The Bio-Rad protein assay kit II (Bio-Rad, Mississauga, ON, Canada) was used to determine the protein concentration based on the Bradford method [27].

Mycotoxins, including DON, NIV, 3-acetyl-DON, 15-acetyl-DON, and chemicals including PQQ, PMS, calcium chloride, trisaminomethane, sodium chloride, acetonitrile, and formic acid, were purchased from Sigma-Aldrich (Oakville, ON, Canada).

### 5.2. DepA Enzyme Activities in Multiple Mycotoxins and Their Metabolites

The initial concentrations of the substrates of DON, NIV, 3-acetyl-DON, and 15-acetyl-DON were 20 µg·mL^−1^. Under the experimental condition (in 250 µL reaction system: DepA: 15 µg·mL^−1^, PQQ: 100 µM, PMS: 40 µM, CaCl_2_: 1 mM, buffer: 50 mM Tris-HCl with 150 mM NaCl, pH = 7.5), a 250 µL sample was collected after 1 h at room temperature (23 °C). The collected samples were immediately added to 100 µL of acetonitrile to terminate the reaction and then added to 150 µL of pure water to make a two-fold dilution. The samples were filtered through 0.45 µm syringe filters before HPLC analysis.

### 5.3. Determination of Kinetics of DepA Enzyme

DON and 15-acetyl-DON with different concentrations ranging from 5 to 100 µg·mL^−1^ were used as substrates of DepA. The 1.0 mL DepA reaction system included 9 µg·mL^−1^ DepA, 100 µM PQQ, 40 µM PMS, 1 mM CaCl_2_, and 50 mM Tris-HCl with 150 mM NaCl (pH = 7.5). The reaction was carried out at room temperature (23 °C), and 50 µL of the sample was collected after 10 min. The reaction was terminated by immediately adding 100 µL of ACN. We added 350 µL of water to make a 1:10 dilution. The samples were filtered through 0.45 µm syringe filters before HPLC analysis. The experimental data were plotted in a line based on the transformation of Michaelis–Menten equation. The maximum reaction rate (V_max_), Michaelis constant (K_m_), turnover number (k_cat_), and catalytic efficiency were calculated and compared.

### 5.4. Effects of Cofactors on DepA Activity in Buffer

In order to evaluate the DepA activity with various enzyme and co-factor concentrations under ideal lab conditions, mathematic models were established in RSM combined with CCRD. The independent variables in the DepA system in buffer included the concentrations of DepA (4.5–13.5 µg·mL^−1^), PQQ (30–90 µM), and PMS (12–36 µM). The initial concentration of DON in the DepA system was 50 µg·mL^−1^. The DepA, cofactors, and DON were mixed to make a 1 mL reaction system with 50 mM Tris-HCl and 150 mM NaCl (pH = 7.5). Design Expert 12.0 was used to perform the statistical design of the experiment and data analysis. A total of 20 runs with 6 replications in the centre points were carried out (Supplemental Table S1). The samples (50 µL) were collected at 20 min. The enzyme reaction from the collected sample was stopped by adding 100 µL of acetonitrile.

### 5.5. HPLC Analysis

Samples and mycotoxin standards were analyzed using Agilent HPLC system (1200 Series, Palo Alto, CA, USA) equipped with a quaternary pump, an inline degasser, and a diode array detector (DAD) with a wavelength set at 218 nm. A Kinetex XB-C18 100 A column (100 × 4.6 mm, 2.6 µm, Phenomenex Inc., Torrance, CA, USA) with a C18-guard column (Torrance, CA, USA) was used for the separation. A binary mobile phase consisting of water (solvent A) and 100% acetonitrile (solvent B) was used. The solvent gradient was 0–80% B in 0–40 min. An extra 10 min of a post-run was added to allow for column restoration. The flow rate was fixed at 0.7 mL min^−1^, while the sample injection volume was set at 10 μL.

### 5.6. Identification of the Transformation Product of 15-Acetyl-DON via LC-MS/MS

LC-MS/MS analysis was performed using a Thermo^®^ Scientific Q-Exactive™ Orbitrap mass spectrometer equipped with a Vanquish™ Flex Binary UPLC System (Waltham, MA, USA). A Kinetex XB-C18 100 A column (100 × 4.6 mm, 2.6 µm, Phenomenex Inc., Torrance, CA, USA) was used. The binary mobile phase consisted of either solvent A (99.9% H_2_O/0.1% formic acid) and solvent B (99.9% acetonitrile/0.1% formic acid) for mass spectrometry analysis or solvent A (100% H_2_O) and solvent B (100% acetonitrile) for UV analysis. The solvent gradients were as follows: 0–40 min, 0% to 80% B; 40–42 min, 80% to 100% B; 42–44 min, 100% B; 44–45 min, 100% to 0% B; 45–51 min, 0% B. The column temperature was set at 23 °C, the flow rate was set at 0.700 mL min^−1^, and the injection volume was set at 10 µL. UV peaks were monitored at 218 nm. The positive mode was used for ion detection, and the spray voltages were set at 3.3 kV. Mass spectrometry data were collected using the Full-MS/DDMS2 (TopN = 10) method, with normalized collision energy (NCE) set at 30 and the intensity threshold set at 1.0 × 10^5^ counts. PRM analysis was performed to confirm the transformation product of 15-acetyl-DON at 10, 20, and 30 NCE. Data were visualized and analysed using Thermo FreeStyle™ 1.7PS2 software, and it was also used to predict the elemental compositions of fragment ions.

### 5.7. Extraction and Purification of the Transformation Product of 15-Acetyl-DON

The mycotoxin 15-acetyl-DON was added into a 20 mL reaction system (DepA: 15 µg·mL^−1^; PQQ: 100 µM; PMS: 40 µM; CaCl_2_: 1 mM; buffer: 50 mM Tris-HCl with 150 mM NaCl; pH = 7.5) with an initial concentration of 100 µg·mL^−1^. After incubation at room temperature for 2 h, the reaction mixture was cleaned and concentrated using OASIS HLB polymeric solid-phase extraction cartridges (150 mg; Waters, Mississauga, ON, Canada) and eluted with 2 mL of acetonitrile. The elute was fractionally collected using the HPLC system mentioned above.

A Phenomenex^®^ 4µ Jupiter Proteo 90 A (250 × 4.6 mm, Phenomenex Inc., Torrance, CA, USA) with a C18-guard column (Torrance, CA, USA) was used for the separation. The compound of interest was eluted using the binary mobile phase at a flow rate of 1.0 mL/min with run time of 20 min. The mobile phase was 40% (*v*/*v*) acetonitrile in water. A volume of 100 µL was injected, and the retention time of the transformation product of 15-acetyl-DON ranged between 12.5 and 14.5 min. The elution fraction was collected using an Agilent 1200 series fraction collector. The collected fraction was dried using a vacuum concentrator (Savant Speedvac SPD1030, ThermoFisher Scientific, Mississauga, ON, Canada), and the pure powder was obtained.

### 5.8. Identification of the Transformation Product 15-Acetyl-DON by NMR

The purified powder was dissolved in 600 µL of dimethyl sulfoxide-d6 (Cambridge Isotope Labs, Tewksbury, MA, USA) and transferred to a 5 mm NMR tube for analysis (Bruker, Billerica, MA, USA). The NMR spectra were recorded at 298 ± 1 K using a 600 MHz Bruker AVANCE III spectrometer equipped with a 5 mm TCI cryoprobe. The ^13^C ^1^H spectrum was collected with 20,480 scans for a total experiment time of 17 h. The edited-HSQC spectrum was collected with 256 increments, 16 scans per increment, and a relaxation delay of 1.5 s, resulting in a total experiment time of 2 h. The HMBC spectrum optimized for an 8 Hz long-range coupling constant was collected with 384 increments, 128 scans per increment, and a relaxation delay of 0.7 s, resulting in a total experiment time of 14.75 h.

### 5.9. Loop Remodelling and DepA-PQQ Complex Docking

The missing loop from the crystal structure of the DepA-PQQ complex from *Devosia albogilva* (PDB: 7WMK) was generated using the SWISS-MODEL online server, as described by Yang et al. [12]. DepA ligand docking was conducted using AutoDock Vina and visualized with PyMOL.

### 5.10. Structure–Activity Relationship Analysis of Different Trichothecenes Complexed with Yeast 80S Ribosomes

The crystal structure data of yeast 80S ribosome complexed with DON (PDB accession number: 4U53) were used for the current analysis. The ligands 3-keto-DON, 3-*epi*-DON, 15-acetyl-DON, and 3-keto-15-acetyl-DON were generated using PyMOL Builder and modelled to create the binding pocket formed by 25S rRNA. Structure models were analyzed and visualized using PyMOL 2.3.4 (www.pymol.org, accessed on 10 February 2024).

### 5.11. Statistical Analysis

DepA activities on DON and 15-acetyl-DON in buffer were performed in triplicate. The kinetics parameters were expressed as mean ± SD (*n* = 3). A one-way analysis of variance (ANOVA), followed by Tukey’s Honest Significant Difference (HSD) test, was performed using SigmaPlot 14.5 (Palo Alto, CA, USA) to analyze the differences in the mean values of the maximum reaction rate (V_max_), Michaelis constant (K_m_), turnover number (k_cat_), and catalytic efficiency of DepA with the substrates of DON and 15-acetyl-DON. The RSM combined with CCRD for the relationship between the DepA reaction rates and the enzyme system were performed using Design Expert 12.0.

## Figures and Tables

**Table 1:** ^1^H and ^13^C NMR chemical shifts of the components to be identified, 15-acetyl-DON, and 3-keto-DON.

	Major Component (Compound 1), ppm		Minor Component (Compound 2), ppm		15-Acetyl-DON, ppm		3-Keto-DON *, ppm	
Site	1H	13C	1H	13C	1H	13C	1H	13C
2	3.54	79.3	3.48	79.6	3.44	79.9	3.53	80.9
3		212.1		213.4	4.22	67.5		212
4	3.04	48.7	3.39	49.5	2.13	43.6	3.13	49.4
4b	2.23		2.13		1.88		2.28	
5		45.6		45.4		45.5		46.4
6		51.2		51.9		51.0		52
7	4.81	72.8	4.75	74.0	4.72	73.3	4.86	74.6
8		198.9		199.6		199.4		199.7
9		136.4		136.0		135.2		135.7
10	6.59	136.6	6.59	136.7	6.59	137.9	6.63	138.3
11	4.51	70.3	4.58	69.9	4.84	69.1	4.82	70.4
12		64.0		64.5		65.4		65.6
13	3.32	47.4	3.28	47.4	3.08	46.5	3.18	47.4
13b	3.2		3.18		2.93		3.09	
14	1.17	13.8	1.21	14.0	0.97	14.1	1.15	14.3
15	4.24	61.7	3.83	60.0	4.25	61.6	3.91	62.5
15b	4.15		3.33		4.08		3.76	
16	1.77	15.0	1.75	14.9	1.76	15.0	1.91	15.3
15-Ac-CH_3_	1.86	20.4			1.84	20.5		
15-Ac-CO		169.6				169.8		

* 3-keto-DON (CDCl_3_) data are derived from Völkl et al., 2004 [11].

**Table 2:** DepA kinetics with the substrates of DON and 15-acetyl-DON. The reaction was performed at 23 °C (pH = 7.5), and the DepA concentration of DepA is 9 µg·mL^−1^.

Substrate	V_max_ (µM·min^−1^)	K_m_ (µM)	kcat (s^−1^)	Catalytic Efficiency (M^−1^s^−1^)
DON	14.79 ± 0.60	221.5 ± 12.7	1.69 ± 0.07	7645 ± 322
15-acetyl-DON	5.99 ± 0.43	107.8 ± 12.1	0.69 ± 0.05	6389 ± 613

## Data Availability

The original contributions presented in the study are included in the article. Further inquiries can be directed to the corresponding author.

## Supplementary Materials

The following supporting information can be downloaded at: https://www.mdpi.com/article/10.3390/toxins16030136/s1, Table S1A: Central composite rotational design matrix for evaluation of co-factors in DepA system; Table S1B: Experimental ranges and levels of independent variables for evaluation of co-factors in DepA system; Table S1C: ANOVA for quadratic model.
