# Supplementary material for: Unveiling the Broad Substrate Specificity of Deoxynivalenol Oxidation Enzyme DepA and Its Role in Detoxifying Trichothecene Mycotoxins
Source: Toxins (Basel). 2024 Mar 5;16(3):136. doi: 10.3390/toxins16030136 (PMC10975547; doi:10.3390/toxins16030136)
Supplement: Supplementary file 1 [file toxins-16-00136-s001.zip › toxins-2861515-supplementary.pdf]

Supplementary Table S1A: Central composite rotational design matrix for evaluation of co-factorsin DepA system

Run	Levels of variable		
	X_1: DepA (μg·mL^{-1})	X_2: PQQ (μM)	X_3: PMS (μM)
1	9.0	60	24
2	13.5	90	36
3	9.0	60	44.2
4	9.0	60	24
5	9.0	60	24
6	4.5	30	12
7	4.5	90	12
8	4.5	90	36
9	9.0	110.5	24
10	13.5	90	12
11	13.5	30	36
12	9.0	60	24
13	4.5	30	36
14	9.0	9.5	24
15	13.5	30	12
16	16.6	60	24
17	9.0	60	24
18	1.4	60	24
19	9.0	60	3.8
20	9.0	60	24

Supplementary Table S1B: Experimental ranges and levels of independent variables for evaluationof co-factors in DepA system

Variables	Symbol	Coded levels				
		-1.682	-1	0	+1	+1.682
DepA (μg·mL^{-1})	X_1	1.4	4.5	9.0	13.5	16.6
PQQ (μM)	X_2	9.5	30	60	90	110.5
PMS (μM)	X_3	3.8	12	24	36	44.2

Supplementary Table S1C: ANOVA for quadratic model

Source	Sum of Squares	df	Mean Square	F-value	p-value
**Model**	**85.74**	**9**	**9.53**	**35.88**	**<0.0001**
X_1 -DepA	43.13	1	43.13	162.40	<0.0001
X_2-PQQ	21.27	1	21.27	80.08	<0.0001
X_3-PMS	12.23	1	12.23	46.04	<0.0001
X_1X_2	0.0490	1	0.0490	0.1844	0.6767
X_1X_3	0.3248	1	0.3248	1.22	0.2946
X_2X_3	0.0219	1	0.0219	0.0824	0.7799
X_1^2	3.03	1	3.03	11.40	0.0070
X_2^2	3.94	1	3.94	14.82	0.0032
X_3^2	3.49	1	3.49	13.15	0.0046
